# Arising Mediastinal Adenocarcinoma in A Patient With A 14 Years History of Mature Teratoma

**DOI:** 10.1002/rcr2.70410

**Published:** 2025-11-14

**Authors:** Yanis Widhiya Ningrum, Isnin Anang Marhana, Farah Fatmawati

**Affiliations:** ^1^ Department of Pulmonology and Respiratory Medicine, Faculty of Medicine Airlangga University Surabaya Indonesia; ^2^ Department of Pulmonology and Respiratory Medicine Dr. Soetomo General Academic Hospital Surabaya Indonesia

**Keywords:** mature teratoma, mediastinal adenocarcinoma, teratoma malignant transformation

## Abstract

Mature teratomas are typically benign germ cell tumours (GCT), but in rare cases, they can undergo malignant transformation. Malignant transformation of a mediastinal teratoma into adenocarcinoma is extremely rare and often poses diagnostic and therapeutic challenges. A 38‐year‐old male with a 14‐year history of a mature mediastinal teratoma, developed a mediastinal adenocarcinoma. The patient initially underwent surgical resection of the teratoma, with no evidence of malignancy at that time. Over the years, the patient remained asymptomatic until the recent onset of cough, prompting further investigation. Imaging studies revealed a recurrent mediastinal mass, and subsequent biopsy confirmed adenocarcinoma arising from the previously diagnosed teratoma. The patient underwent debulking thoracotomy and chemotherapy. This case highlights the importance of long‐term follow‐up in patients with mediastinal teratomas, given the rare but significant risk of malignant transformation. Awareness of this possibility can facilitate early diagnosis and timely intervention, ultimately improving patient outcomes.

## Graphical abstract

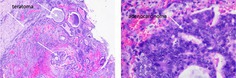

## 1 Introduction

The most common types of mediastinal tumours in adult patients are thymoma and thymic cyst (26.5%) and neurogenic tumours (20.2%), followed by germ cell tumour (GCT) (13.8%), lymphoma (12.7%), foregut cyst (10.3%) and pleuropericardial cyst (6.6%) [1, 2]. Mature teratoma mediastinal is a benign GCT in the mediastinum. Matured teratomas show well‐differentiated somatic elements, such as nerves, fat, skin and cartilage [3].

Teratoma with Malignant Transformation (TMT) has been classified into two clinical and pathological types: first, TMT that is caused by chemotherapy or irradiation and second, naturally occurring TMT [4]. Almost all cases of TMT are of the first type, and tend to occur in young patients with early symptoms of malignant GCT. The second type is rarely reported [5]. A review of the literature revealed that there were only nine reports of naturally occurring mediastinal TMT with accurate pathology description, and seven of them were adenocarcinoma type. Adenocarcinoma is likely the most frequent histology of somatic‐type malignancies arising from naturally occurring TMT [6, 7, 8, 9].

The mechanism of teratoma transformation to malignancy is still largely unknown. One of the journals that reported the malignant transformation of teratoma in the ovary, mentioned that malignant transformation of teratoma is the result of progressive accumulation of genetic mutations [9]. Somatic‐type malignancies are thought to develop from the malignant transformation of pre‐existing teratomatous elements or differentiation from totipotent germ cells. Epithelial malignancies associated with GCT are mostly of the adenocarcinoma type [10].

Given the rarity of somatic malignancies, the pathogenesis of this disease remains difficult to study. One hypothesis is that the emergence of somatic malignancies is the result of the transformation of the teratomatous component of GCTs; another hypothesis states that somatic‐type malignancies and GCTs are clonally related and derived from pluripotent progenitor cells. This has been studied by Kum et al. who studied 27 cases of teratomas with somatic‐type malignancies in metastatic lesions, and showed that somatic‐type malignancies in GCTs have the same genetic aberrations as in teratomas of origin [11].

## 2 Case Report

**FIGURE 1:**
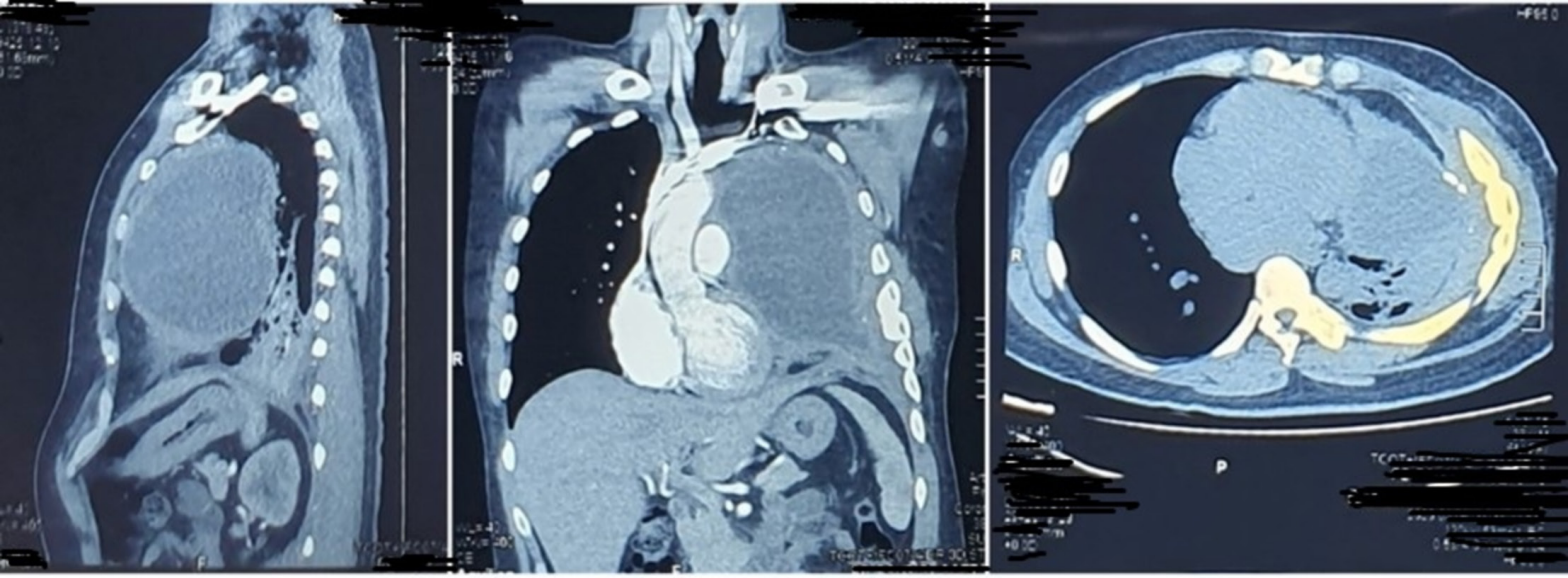
A predominantly cystic mass (23 HU) with solid (43 HU), fat (−32 HU), calcified (207 HU) components, well‐defined borders, regular edges in the anteromedius mediastinum, size ±10.79 × 12.43 × 13.59 cm, which on contrast administration shows contrast enhancement (87 HU) on its walls.

A 38‐year‐old male presented with a chronic cough lasting for 1 year, which had worsened over the past month, accompanied by a weight loss of approximately 10 kg within a year. Fourteen years earlier, he had been diagnosed with a mediastinal teratoma and underwent tumour excision, leaving part of the capsule attached to surrounding tissue. A previous CT scan showed a well‐defined mass in the anteromedial mediastinum consistent with a mature teratoma (Figure 1).

**FIGURE 2:**
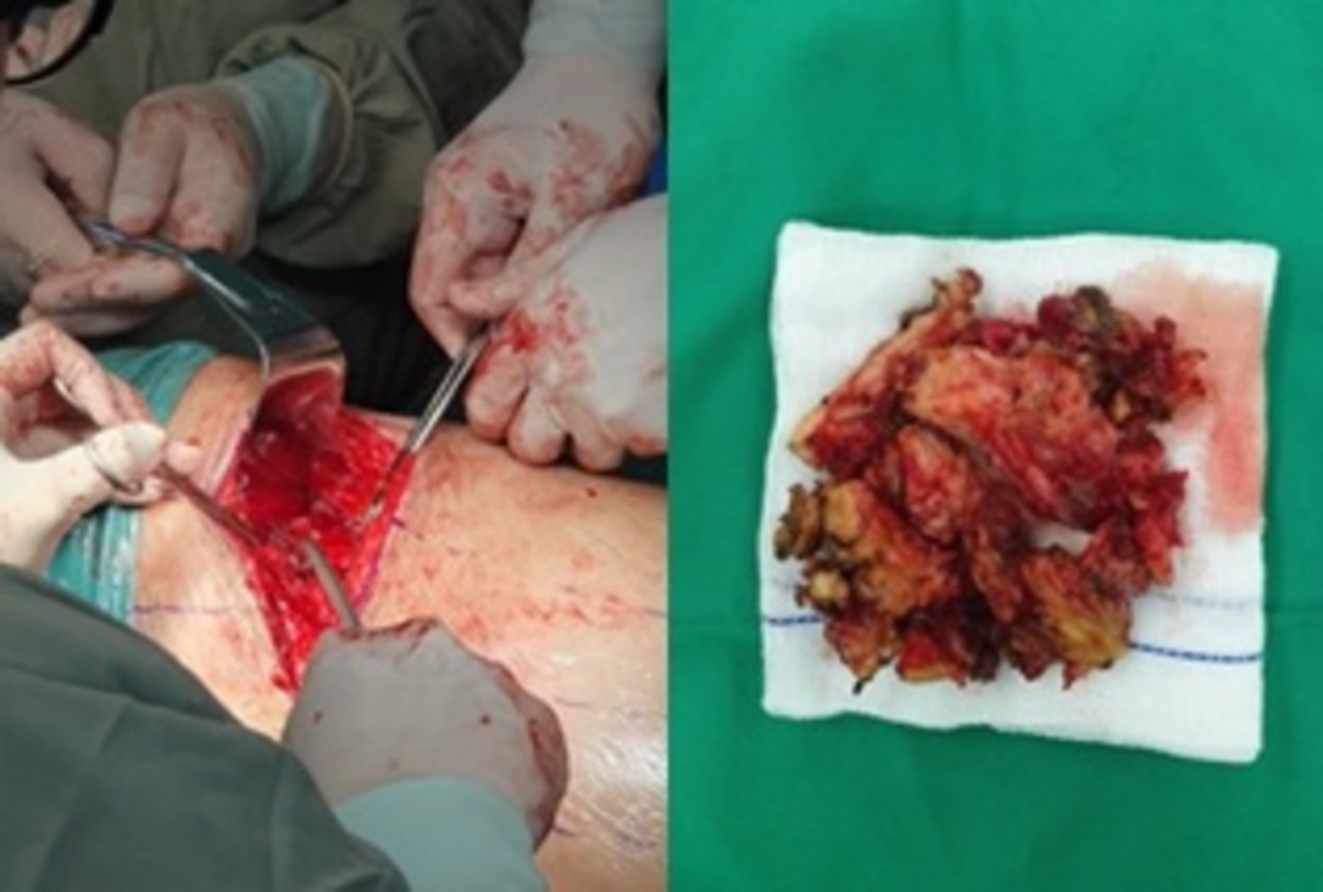
Thoracotomy operation, debulking mediastinal tumours.

**FIGURE 3:**
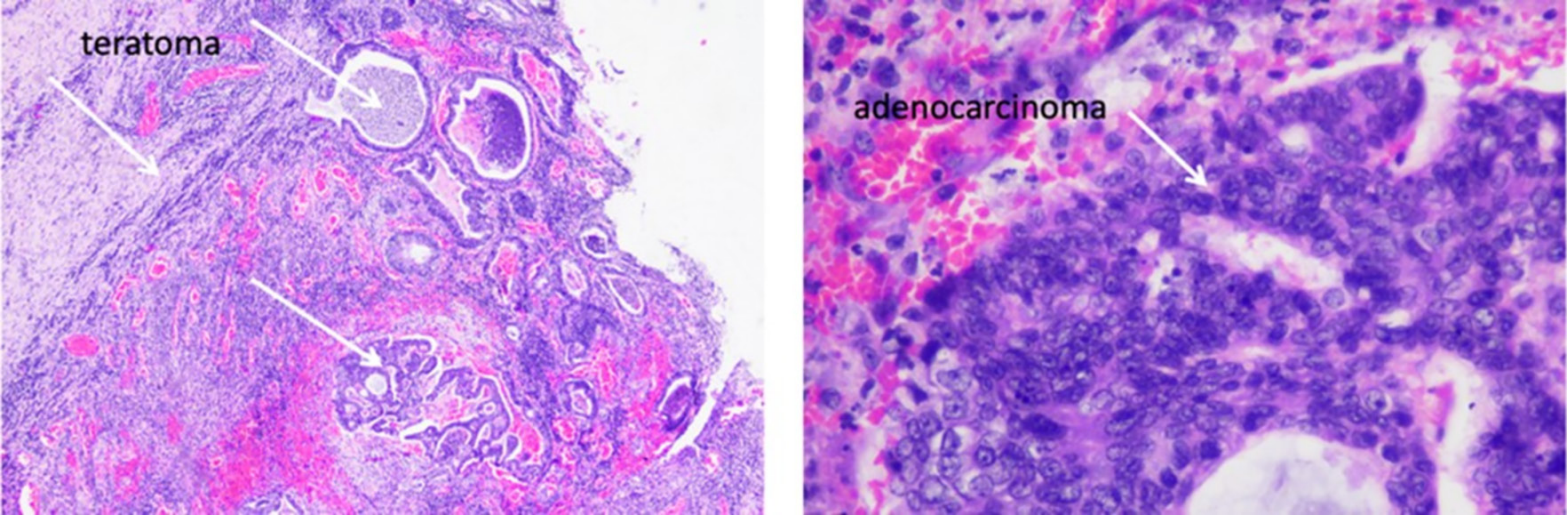
Histo pathology anatomy of adenocarcinoma growing from teratoma.

A multidisciplinary tumour board recommended an open biopsy and exploratory thoracotomy. Intraoperatively, a fistulous tract extending to the skin was identified and excised, with evacuation of approximately 500 mL of purulent fluid. The tumour was adherent to the chest wall, and an incisional biopsy was performed (Figure 2). Histopathology revealed a well‐differentiated adenocarcinoma of mediastinal origin, suggesting malignant transformation of a pre‐existing teratoma (Figure 3).

Subsequent tumour board discussion led to a decision for debulking thoracotomy surgery of the left mediastinal tumour. Intraoperatively, a solid mediastinal mass occupying the left hemithorax and firmly adherent to the chest wall and intercostal spaces was observed. The procedure was terminated due to excessive bleeding. Immunohistochemistry showed Napsin A positivity and TTF‐1 negativity, confirming adenocarcinoma that was unlikely to be pulmonary in origin. The patient subsequently received a paclitaxel–carboplatin chemotherapy regimen.

## 3 Discussion

Somatic malignancy arising from a GCT is a rare phenomenon, occurring in approximately 2.7%–8.6% of GCT cases, and is more commonly observed in late relapse cases [11]. In 2017 several studies have been collected; there were 21 cases reported of mediastinal mature teratoma with malignant transformation in adenocarcinoma in the literature. Fourteen males, six females and one uncharacterised patient were identified with a median age of 39 years old. Six of them were asymptomatic at the time of the initial diagnosis. In 18 cases, the malignant transformation in adenocarcinoma was diagnosed simultaneously with the mediastinal mature teratoma while in three cases the mediastinal mature teratoma predated the adenocarcinoma transformation with a range of 6 months–14 years [12]. The diagnosis of malignant transformation was primarily based on clinical findings. In patients with a history of treated or suspected mature teratoma, worsening or changes in clinical symptoms often led to the identification of transformation [12]. In this case report, the patient has a cough and a decrease in body weight that's been ongoing for a year prior to diagnosis.

The diagnosis of teratoma transformation malignancy is generally preceded by clinical findings. In patients with previously treated or suspected mature teratoma, exacerbation or worsening of clinical signs usually leads to the diagnosis of malignant transformation. Two studies reported patients who were diagnosed with mature teratoma with malignant transformation due to haemoptysis and weight loss, 7 and 20 years after the radiological diagnosis of asymptomatic mediastinal tumours [4, 8, 12]. In this case report, the patient has a cough and a decrease in body weight that's been ongoing for a year prior to diagnosis. After the CT scan, the patient was found to have a mature teratoma in the anteromedius mediastinum measuring ±10.79 × 12.43 × 13.59 cm with complex rupture to the cutaneous area in the sternum region. Then to confirm the diagnosis, open biopsy and tumour excision were performed with the biopsy result of adenocarcinoma growing from the teratoma.

A previous study suggested that mature teratoma cells can differentiate into malignant tissue in vitro, and this phenomenon appears after specific chromosomal changes in TMT. In a study at Memorial Sloan Kettering Center, chromosome 12 abnormalities were found in 11 out of 12 TMT cases, of which 10 were i(12p) [13, 14]. The discovery of isochromosome 12p (i(12p)), which is a specific marker for the GCT component among non‐GCTs in TMT, can help make the diagnosis [15].

The likelihood of curing GCT patients with TMT largely depends on the resectability of the disease. Some single chemotherapeutic agents (i.e., ifosfamide or paclitaxel) have activity in GCT and TMT. Adjuvant chemotherapy preceded by resection had no impact on overall survival, as four patients with TMT adenocarcinoma who underwent complete resection without adjuvant chemotherapy remained recurrence‐free during follow‐up. Significant influences on overall survival were histologic type and stage of the cancer. The best overall survival was for rhabdomyosarcoma transformation type, followed by NAS (not otherwise specified) sarcoma, other and mixed types, adenocarcinoma and lastly PNET (Primitive Neuroectodermal Tumour) while for stage, the higher the stage the lower the overall survival [16].

In a case report by Agarwal et al., histology‐based tailored chemotherapy—such as a 5‐fluorouracil‐based regimen for adenocarcinoma transformation—was recently recommended, as it may improve the outcomes of GCT patients with teratoma with malignant transformation (TMT) [7]. Another study reported a patient who received paclitaxel (200 mg/m^2^) and carboplatin (AUC 6) every three weeks; however, the disease gradually progressed despite treatment [6]. In our case, the patient was treated with a paclitaxel (100 mg/m^2^)–carboplatin (AUC 5) regimen administered every 3 weeks for six cycles, resulting in stable disease, with ongoing follow‐up planned for the next 3 months [17].

Teratomas that transform to malignancy have a poor prognosis. Transformation to malignancy has a poor impact on the survival rate, especially for those diagnosed after therapy. The prognosis is even worse if metastasis is found in any organ. In a study conducted by Giannatempo et al. the 5‐year survival rate in patients with metastases varied between 47.9% and 69.8%. Adjuvant chemotherapy had no impact on the overall survival (OS) observed. TMT progression appears to have a detrimental impact on OS regardless of clinical stage and International Germ Cell Cancer Collaborative Group (IGCCG) prognostic category. The 5‐year OS for patients with clinical stage I was 83.4%, and 69.8% for metastatic patients with good prognosis, 49.1% for metastatic patients with moderate prognosis, and 47.9% for metastatic patients with poor prognosis. TMT found after previous GCT treatment is associated with lower OS, especially when TMT has been diagnosed more than 2 years afterward [16].

The prognosis of patients with mature teratomas with TMT, which are resected completely, appears to be excellent. However, incomplete resection of the tumour is likely to be associated with a poor prognosis [6, 8]. Teratomas with TMT are conventionally considered unresponsive to chemotherapy even though the choice of chemotherapy has been based on the histology of the transformation [6, 17]. It is important to identify the malignant component in teratomas, due to the ability of malignant cells to infiltrate into adjacent organs or even metastasize, thereby worsening disease progression. Survival rates are highly dependent on the completeness of the resection and the severity of the disease [17]. In this case, incomplete resection was performed 14 years prior leaving the capsule due to adhesion. The patient underwent chemotherapy based on the results of TMT adenocarcinoma, with a paclitaxel‐carboplatin regimen. Although there is currently no metastasis in this patient and no progressivity, the prognosis is unfavourable based on previous studies.

In conclusion, the mechanism of teratoma transformation to malignancy is still largely unknown. This disease is still relatively rare and is still being studied. Complete resection is an essential therapy when the tumour is confined to a single site. For patients with TMT, chemotherapy based on adenocarcinoma transformation may help improve patient outcome. In our case, a patient with TMT adenocarcinoma relapsed after 14 years post tumour resection. The patient underwent incomplete resection and received six cycles of chemotherapy. The prognosis of mature teratoma patients with complete resection of TMT seems to be very good. However, incomplete tumour resection is most likely associated with poor prognosis.

## Author Contributions

**Yanis Widhiya Ningrum:** patient evaluation, data collection, manuscript drafting. **Isnin Anang Marhana:** image selection, writing, review and editing, final approval of the manuscript. **Farah Fatmawati:** references, writing – review and editing, final approval of the manuscript.

## Consent

The authors declare that written informed consent was obtained for the publication of this manuscript and accompanying images using the consent form provided by the Journal.

## Conflicts of Interest

The authors declare no conflicts of interest.

## Data Availability

The data that support the findings of this study are available from the corresponding author upon reasonable request.
